# Spatial Analysis of Soil Organic Carbon in Zhifanggou Catchment of the Loess Plateau

**DOI:** 10.1371/journal.pone.0083061

**Published:** 2013-12-27

**Authors:** Mingming Li, Xingchang Zhang, Qing Zhen, Fengpeng Han

**Affiliations:** 1 Institute of Soil and Water Conservation, Chinese Academy of Sciences and Ministry of Water Resources, Yangling, PR China; 2 University of Chinese Academy of Sciences, Beijing, PR China; 3 State Key Laboratory of Soil Erosion and Dryland Farming on the Loess Plateau, Institute of Soil and Water Conservation,Northwest A & F University, Yangling, PR China; DOE Pacific Northwest National Laboratory, United States of America

## Abstract

Soil organic carbon (SOC) reflects soil quality and plays a critical role in soil protection, food safety, and global climate changes. This study involved grid sampling at different depths (6 layers) between 0 and 100 cm in a catchment. A total of 1282 soil samples were collected from 215 plots over 8.27 km^2^. A combination of conventional analytical methods and geostatistical methods were used to analyze the data for spatial variability and soil carbon content patterns. The mean SOC content in the 1282 samples from the study field was 3.08 g·kg^−1^. The SOC content of each layer decreased with increasing soil depth by a power function relationship. The SOC content of each layer was moderately variable and followed a lognormal distribution. The semi-variograms of the SOC contents of the six different layers were fit with the following models: exponential, spherical, exponential, Gaussian, exponential, and exponential, respectively. A moderate spatial dependence was observed in the 0–10 and 10–20 cm layers, which resulted from stochastic and structural factors. The spatial distribution of SOC content in the four layers between 20 and 100 cm exhibit were mainly restricted by structural factors. Correlations within each layer were observed between 234 and 562 m. A classical Kriging interpolation was used to directly visualize the spatial distribution of SOC in the catchment. The variability in spatial distribution was related to topography, land use type, and human activity. Finally, the vertical distribution of SOC decreased. Our results suggest that the ordinary Kriging interpolation can directly reveal the spatial distribution of SOC and the sample distance about this study is sufficient for interpolation or plotting. More research is needed, however, to clarify the spatial variability on the bigger scale and better understand the factors controlling spatial variability of soil carbon in the Loess Plateau region.

## Introduction

Soil organic carbon (SOC) is an important aspect of soil quality and plays an important role in soil productivity, environmental protection, and food safety [1]. Because SOC is the biggest part of the terrestrial carbon cycle and carbon-based greenhouse gas balance research [2], slight changes in SOC can greatly impact atmospheric CO_2_ concentrations and global climate change. Therefore, SOC has become a core topic in global climate change research. Considerable attention has focused on SOC in relation to climate change and greenhouse gas emissions [3], [4].

The SOC has a strong spatial heterogeneity which can be expressed by a function [5], [6]. A precise understanding of SOC spatial characteristics can improve the accuracy of SOC stock estimations and contribute to the development and implementation of effective carbon sequestration methods. Recently, a series of studies regarding SOC spatial distribution and stock were conducted by international researchers. ie., in some European countries [7], [8], the United States [9], India [10], Brazil [11], and other countries. These studies indicated that the spatial variability of SOC characteristics was affected by multiple factors, including land use, soil parent material, topography, vegetation, climate, and agricultural use [12]–[15].

The Loess Plateau of China is located in an ecologically vulnerable semi-arid region that is affected by one of the most serious soil erosion problems in the world. In the past decade, large-scale vegetation recovery and ecosystem improvement (to a certain extent) have occurred as a result of the “Grain for Green Project” implemented by the Chinese government [16]. Due to its complex and broken topography and hilly and gully landforms, spatial heterogeneity in the Loess Plateau region is relatively high [17]. Although many studies have been conducted, the data in these studies were mainly collected at slope and [18], [19] ecosystem scales [20], [21] and from shallow soil layers [22]–[24]. In addition, SOC spatial variability studies at a catchment scale have mainly focused on the environmental features that resulted from different land uses and soil types [25]–[27]. These SOC measurements were rarely related to the depth of the soil layers. Generally, only small amounts of data were used in these analyses, due to the considerable effort required to obtain data in this complex terrain. Many of the studies mentioned above are associated with significant uncertainty. This uncertainty results from the unavailability of complete data sets, the diversity of the data sources, and the inherent spatial heterogeneity of the SOC [28].

Two objectives were addressed in this study: 1) obtaining the vertical distribution of SOC in a typical Loess Plateau small catchment; 2) elucidating the spatial variability and distribution of SOC at different depths within the catchment.

## Materials and Methods

### Study area

**Figure 1:**
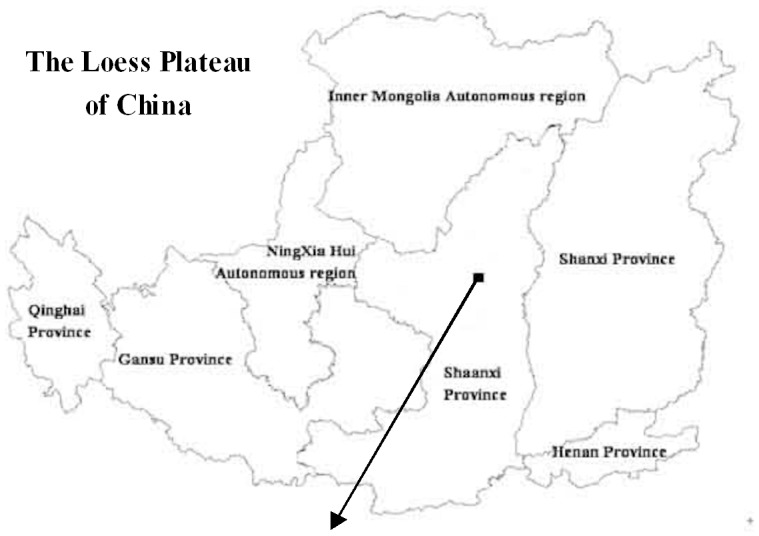
The location of the catchment.

**Figure 2:**
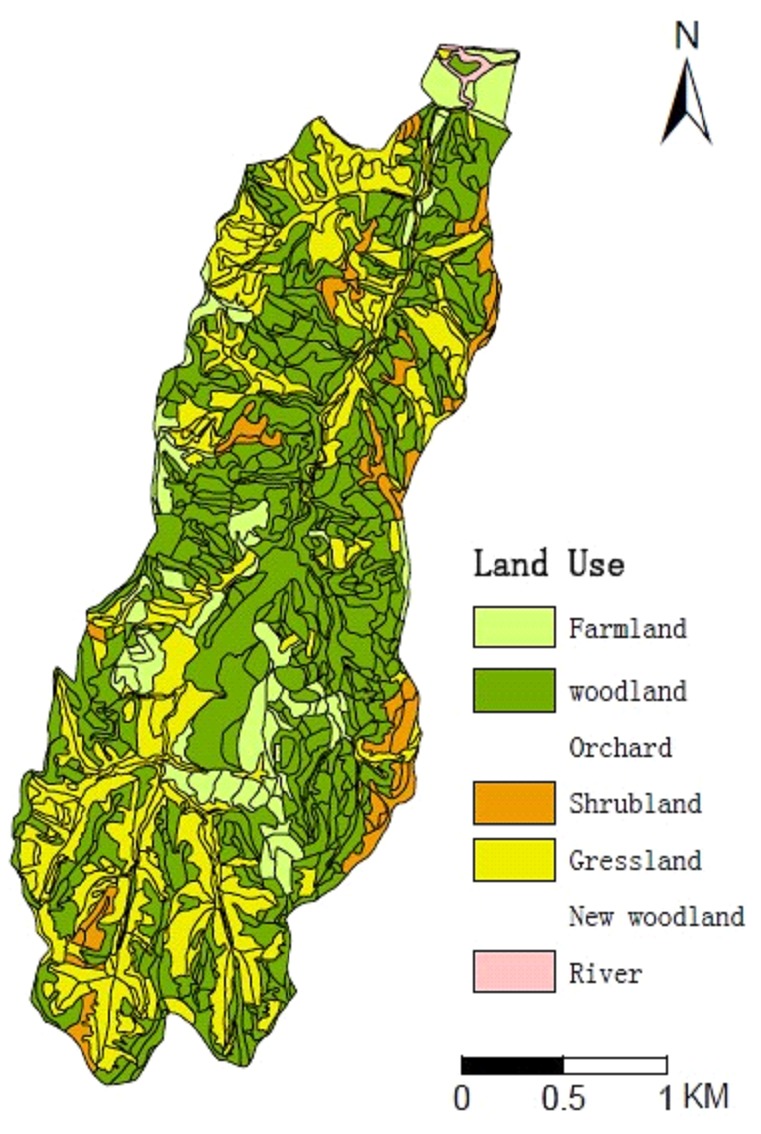
The land use types.

The Zhifanggou catchment is a typical small catchment on the Loess Plateau. which is located in Ansai County, Shaanxi Province, China (longitude 108°51′44″−109°26′18″, latitude 36°30′45″−37°9′31″, altitude 1,010″−1,1431 m, 8.27 km^2^) (Fig. 1). The geomorphology of this catchment is extremely broken and exhibits the characteristics of a valley. The soils are predominantly loess and uniform in texture. The sand, silt, and clay contents of the soil are 65, 24, and 11%, respectively. The average annual precipitation in the catchment is 541.2 mm. In addition, 75% percent of the annual rainfall in this region occurs between July and September. During these months, the rainfall is intense and causes extensive erosion. The study area is under four main land use types that cover woodland (54%), grassland (32%), farmland (8%)and shrubland (6%). (Fig. 2). The main land uses (and vegetation species) are shrubland(*r*), woodland *(Populous simonii Carr., Fruit trees*) grassland (*Medicago sativa L., Artemisia gmelinii, Stipa bungeana, Artemisia scoparia,*) and farmland (*Triticum aestivum, Zea mays, Glycine max*) [29].

### Sampling method

**Figure 3:**
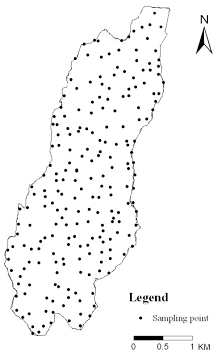
The locations of the sampling.

The grid method was used to collect soil samples. All of the designated sample sites were arranged on a 1∶10,000 scale topographic map. A grid interval of 200×200 m was used, and each grid was considered an independent study unit. A portable GPS was used to locate each sample site. Each site was divided into 6 depths between 0–100 cm as follows: 0–10, 10–20, 20–40, 40–60, 60–80 and 80–100 cm. All samples were collected with a 5-cm-diameter hand auger. 215 soil sampling sites including farmland 28, shrubland 33, woodland 77 and grassland 77. A total of 1,282 soil samples were collected from 215 soil sampling sites (Fig. 3). Soil samples were air dried before passage through a 0.25 mm sieve for laboratory analysis. The SOC content of each sample was determined in duplicate with the dichromate oxidation (external heat applied) method [30]. The samples were collected in November 2010.

### Data processing and analysis

The geostatistical method is a spatial analysis method that was developed from classical statistics. Based on the theory of regionalized variables, this method effectively uses semi-variogram and Kriging interpolations to determine the spatial distribution, variability, and related characteristics of the various random structural variables [31]. The semi-variance function was fit based on the coefficient of determination R^2^ and the residual sum of squares (RSS) to obtain an optimal theoretical mode [32].

The Kriging interpolation method was used to estimate the values of the unmeasured sites *x_0_* by assuming that *z*' *(x_0_)* equals the linear sum of the known measured values. This process is expressed by the following equation [33]:
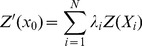
(1)where *Z*' *(x_0_)* is the predicted value at position *x_0_*_,_ Z(*X_i_*) is the known value at sampling site *X_i_*. *λ_i_* is the weighting coefficient of the measured site. and *N* is the number of sites within the neighborhood searched for the interpolation.

The data that were used in this study were analyzed with classical statistical methods in the program SPSS 18.0. Analysis of variance (ANOVA) was performed with the least significant difference (LSD) method to compare the impacts of different soil depths on SOC content (P<0.05). The K-S (Kolmogorov-Smirnov) test was used to determine if the data were normally distributed. Logarithmic or other transformations were performed on data that were not normally distributed to obtain a normal distribution. The use of non-normally distributed data would increase the estimation of error. Therefore, it was necessary to transform these non-normally distributed data. The test results indicated that the SOC distributions were skewed at soil depths of 10–20, 20–40, 40–60, and 80–100 cm and were normal at soil depths of 0–10 and 60–80 cm. However, the normally distributed SOC contents were highly skewed and had a high kurtosis. Thus, a logarithmic conversion of the SOC contents of the six soil layers was performed. The kurtosis and skewness of the SOC content decreased in each soil layer and were normally distributed. After logarithmic conversion, the normally distributed data were imported into the software GS +9.0 for semi-variance fitting., GS +9.0 software was used to obtain semi-variance fits and an optimal theoretical model. The ArcGIS9.3 software was used for the classical Kriging interpolation and for plotting the spatial distribution.

## Results and Discussion

### Descriptive statistics of the SOC content

The descriptive statistics obtained from SOC in the study area are presented in Table 1. The mean SOC content was 3.08 g·kg^−1^ in the study area, well below the average SOC level in China [34]. The mean SOC of all soil layers was between 2.20 and 6.36 g·kg^−1^. The highest SOC content in the study area was observed in the 0–10 cm layer. The SOC content decreased with increasing soil depth. As anticipated, the lowest SOC content was observed at a depth of 80–100 cm because SOC is mainly formed by the decomposition of animal and plant residues that are primarily distributed in the soil surface and decrease with depth. The higher SOC in the surface soils indicates that the surface soil actively participates in “carbon sequestration”. The relationship between SOC content (unit: g/kg) and soil depth (unit: cm) is expressed by the following power function: y = 17.501*x*−0.462, R2 = 0.9889, *p*<0.001. These results are similar to those reported by [17].

**Table 1:** Summary statistics from the classical analyses of soil organic carbon (SOC) content.

Soil Depth(cm)	*N*	Mean (g·kg−^1^)	Median (g·kg−^1^)	Min (g·kg−^1^)	Max (g·kg−^1^)	Std.D.	C.V.(%)	Skewness	Kurtosis	Distribution type
0–10	215	6.36^a^	5.09	1.30	30.22	3.96	62	2.30	7.40	NN
								0.51	0.54	N^log^ *
10–20	215	4.43^b^	3.84	1.33	14.87	2.12	48	1.62	3.21	n
								0.36	0.12	N^log^ *
20–40	215	2.99^c^	2.58	0.95	8.78	1.41	47	1.74	3.42	n
								0.50	0.34	N^log^ *
40–60	215	2.49^d^	2.17	1.00	6.69	1.10	44	1.70	3.14	n
								0.61	0.23	N^log^ *
60–80	213	2.29^d^	2.01	0.66	11.69	1.14	50	1.96	5.42	NN
								0.51	0.93	N^log^ *
80–100	209	2.20^e^	1.96	0.25	8.51	1.03	47	1.50	3.64	n
								0.17	0.95	N^log^ *

*Notes: N*., Number of samples; *C.V.*, Coefficient of Variation; *Std. D.*, Standard Deviation.

*a, b, c, d, e,* Different lowercase letters represent a significant difference between the layers (*P*<0.05).

, Natural logarithm transformation with the corresponding skewness and kurtosis values.

*N*, Normal distribution; *n*, Near Normal Distribution; *NN,* Non-Normal Distribution; *N^log^*, Log-Normal Distribution.

The SOC coefficients of variation in the six layers were 62, 48, 47, 44, 50, and 47%, respectively. According to the classification system proposed by Nielson and Bouma [35], the variable is considered to have weak variability if the coefficient of variation (CV) is less than 10% and moderate variability if the CV is between 10% and 100%; otherwise, the variable has strong variability. Therefore, these values all correspond to moderate variability. The highest coefficient of variation was 62%, at a soil depth of 0–10 cm. The lowest coefficient of variation was 44%, at a soil depth of 40–60 cm. This low coefficient of variation resulted from the influence of multiple factors on the soil surface, including human intervention, vegetation type, land use, and topography. The average SOC contents were significantly different for each soil layer. This finding indicated that the central tendency of the SOC distribution was likely affected by anomalous values that led to a non-normal distribution.

### Geostatistical analysis of the SOC contents

A table of SOC variability characteristics was generated from semi-variance fitting (Table 2). In Table 2, C_0_ is the nugget variance, C is the structural variance, and C_0_+C is the sill. C_0_/C_0_+C represents the degree of spatial variability, which is affected by both structural and stochastic factors. Higher ratios indicate that the spatial variability is primarily caused by stochastic factors, such as fertilization, farming measures, cropping systems, and other human activities. By contrast, a lower ratio suggests that structural factors, such as climate, parent material, topography, soil texture, soil type, and other natural factors, play a significant role in spatial variability. In addition, a proportion less than 25% indicates a strong spatial correlation in the system, a proportion between 25% and 75% indicates a moderate spatial correlation, and a proportion larger than 75% indicates a weak spatial correlation. If the proportion is near 1, then the variable is constant at all scales [36].

**Table 2:** Geostatistical parameters for soil organic carbon(SOC) content.

Soil Depth(cm)	Model	C_0_	C_0_+C	Proportion (C_0_/C_0_+C)	Range (m)	R^2^	RSS
0–10	Exponential	0.1306	0.2832	0.461	552	0.822	3.036E-03
10–20	Spherical	0.0968	0.1946	0.497	562	0.907	8.505E-04
20–40	Exponential	0.0314	0.1788	0.176	234	0.682	1.121E-03
40–60	Gaussian	0.0201	0.1472	0.137	233	0.915	1.240E-03
60–80	Exponential	0.0249	0.1348	0.185	254	0.533	1.354E-03
80–100	Gaussian	0.0204	0.1358	0.150	264	0.774	2.733E-04

As shown in Table 2, the C_0_/C_0_+C values for SOC were 0.461, 0.497, 0.176, 0.137, 0.185, and 0.150, respectively, in the six different soil layers. The proportion was between 25 and 75% at soil depths of 0–10 and 10–20 cm, indicating a moderate spatial correlation. This correlation was apparent in the 552 and 562 m ranges, respectively, and was subjected to the impacts of stochastic and structural factors. The C_0_/C_0_+C was less than 25% in the four layers at a depth of 20 to 100 cm, indicating a strong spatial correlation. This spatial correlation was apparent in the 414, 234, 534, and 264 m ranges and was affected by structural factors. The spatial correlation ranges are different from Han [17] and Liu [15] that are caused by the different study area.

The variability range determines the spatial autocorrelation. When a variable is within the range values, it is spatially autocorrelated, and when it is outside of the range values, it is not. This determination provides guidelines for effectively designing sampling schemes [26]. In this study, large range variations occurred between 234 and 562 m that is to say during the large range the data have the spatial autocorrelation. In general, the sampling distances that are outside of the range are invalid for interpolation or plotting [37]. The average sampling grid interval was 200 m in this study. This sampling grid was smaller than the minimal range of 234 m, which indicates that the sampling interval in the study area met the requirements for spatial variability analysis.

**Figure 4:**
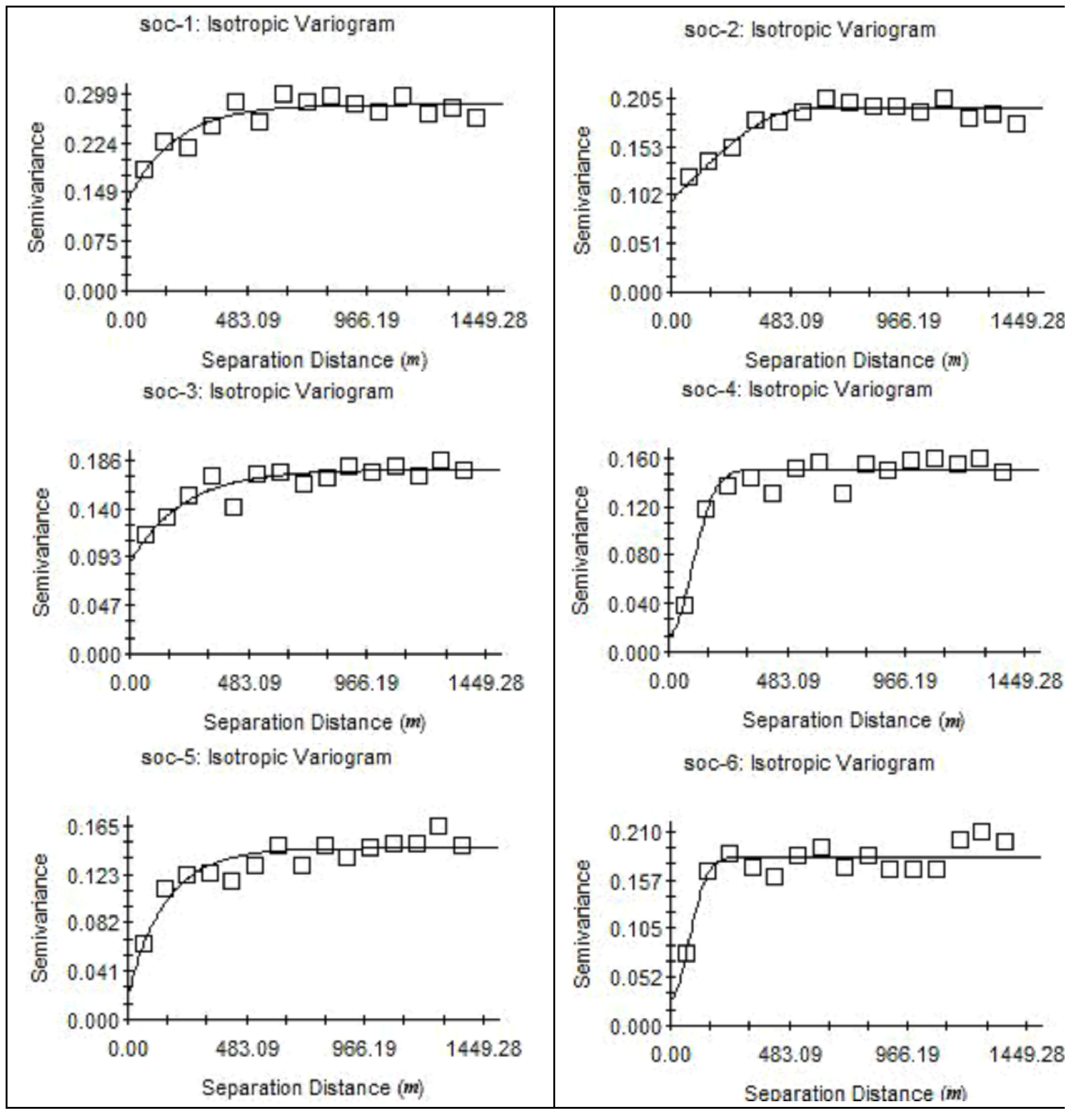
Semi-variance charts of soil organic carbon (SOC) under different soil depths.

The semi-variance function model fitting curve for each soil layer was obtained using the semi-variance function. The semi-variance function of the SOC in the soil layers displayed the same trend (Fig. 4). The function values gradually increased with increasing spatial distance before stabilizing. The semi-variogram of the SOC contents at depths of 0–10, 10–20, 20–40, 40–60, 60–80, and 80–100 cm corresponded with the following models: exponential, spherical, exponential, Gaussian, exponential, and Gaussian, respectively. All six layers had coefficients of determination R2 of 0.682 to 0.915 and a small RSS. These results indicate that the theoretical model was an adequate representation of the spatial structural characteristics of the SOC contents in the soil layers. In addition, the curve fit for each layer was optimized.

### Spatial distribution of the SOC content

**Figure 5:**
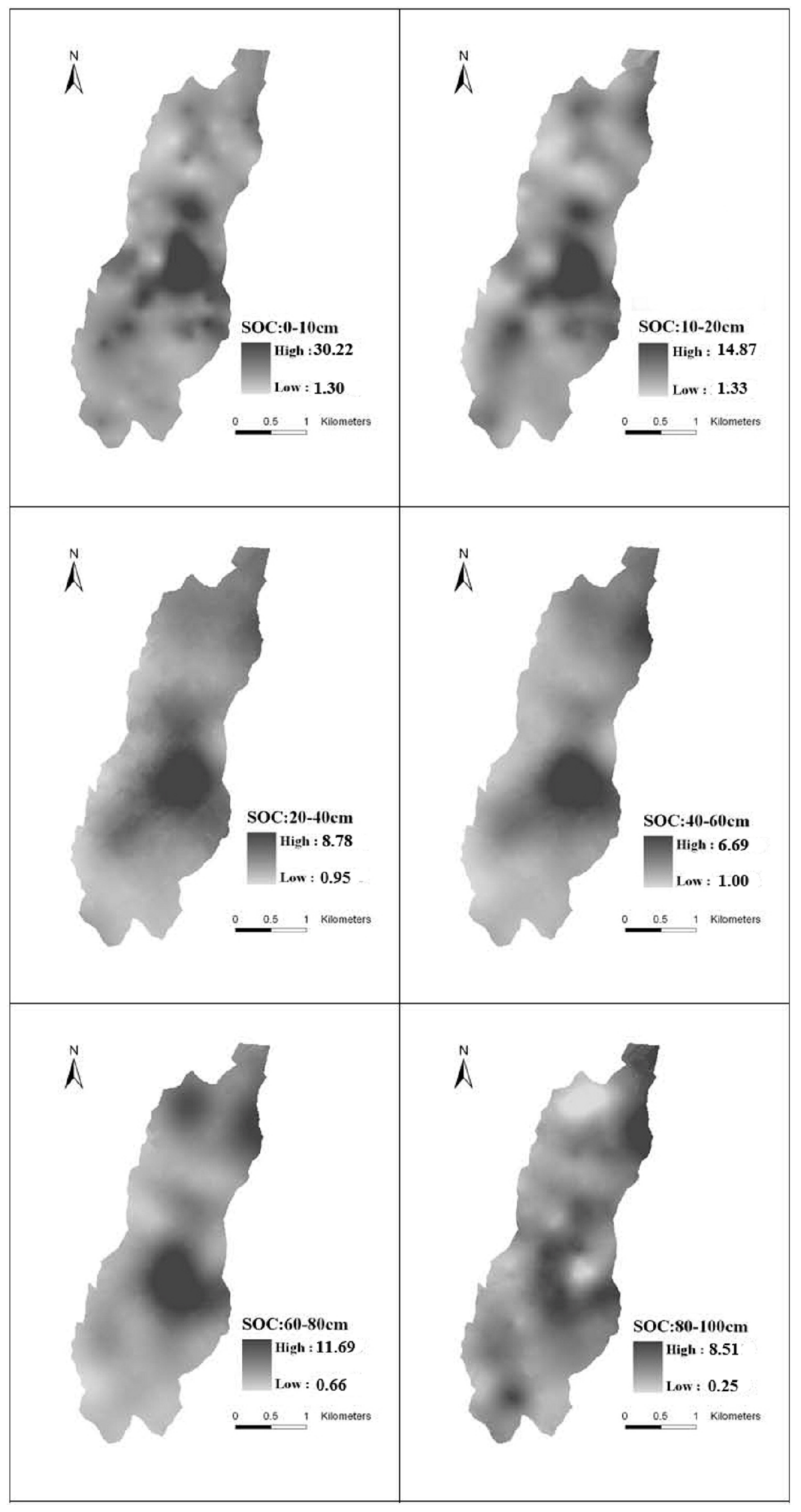
The spatial distribution of the soil organic carbon (SOC)under different soil depths in the catchment.

To visualize directly the spatial distribution of SOC content in this catchment (according to the obtained semi-variogram model), the ordinary Kriging interpolation method from geostatistics was adopted to interpolate each layer in the study area and to generate a spatial distribution diagram of SOC content (Fig. 5).

As shown in Fig. 5, the overall spatial distribution of SOC density in each layer was observed in patches or speckles. Previous studies have shown that the distributions of SOC contents in soils result from the combined effects of soil parent material, climate, topography, landscape, and human intervention [38]. In this study, the catchment area was small with a uniform climate, soil parent material, and soil type. Consequently, the SOC content variations were only related to the landscape and human activities.

**Figure 6:**
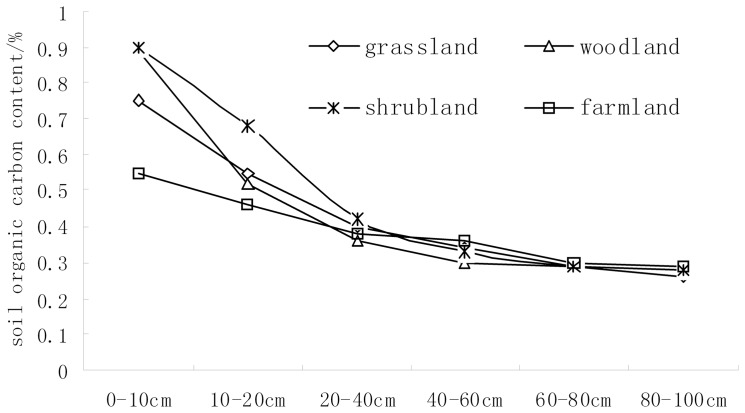
The soil organic content in the different land use types.

Figures 2 and 5 depict areas with significantly high SOC content in each layer in the mid-east and north regions of the catchment. These areas are mainly covered by woodland and fruit trees. The gully channels also contained high concentrations of SOC. The SOC content of the peripheral areas of the catchment was lower due to their higher elevation. From the vertical direction, the 0–10 and 10–20 cm depths had smaller spot areas with dispersed distributions, indicating strong variability. The highest-content spots occurred in the woodlands and shrub lands. By contrast, the lowest-content spots occurred in the grasslands and farmlands. That is to say the woodland and shrub lands can increase the soil organic carbon content. Form Fig 6 we can know that the SOC content in the 0–10 and 10–20 cm depths was shrub lands > woodlands > grasslands > farmlands. No significant variations in SOC spatial distributions were observed in the other four soil layers that had concentrated and high-content areas. The 80–100 cm depth had loosely distributed spots in which the low-content spots corresponded with grasslands and farmlands. Therefore, topographical factors, land use, and human activities were the major causes of spatial variability in SOC distribution. In addition, the ordinary Kriging interpolation directly reflected the spatial distribution of SOC in this catchment.

## Conclusions

This study showed that the overall spatial distribution of the SOC density in each layer of the study area was observed in patches or speckles and the coefficient of variation of the SOC content in each layer was moderate variability. Correlations within each layer were observed between 234 and 562 m. Our results suggest that the ordinary Kriging interpolation can directly reveal the spatial distribution of SOC and the sample distance about this study is sufficient for interpolation or plotting. More research is needed, however, to clarify the spatial variability on the bigger scale and better understand the factors controlling spatial variability of soil carbon in the Loess Plateau region.
