# Correction: Spatial Analysis of Soil Organic Carbon in Zhifanggou Catchment of the Loess Plateau

**DOI:** 10.1371/annotation/c81daa7c-5375-4349-970b-c63d288947eb

**Published:** 2014-01-09

**Authors:** Mingming Li, Xingchang Zhang, Qing Zhen, Fengpeng Han

The order of affiliations for all authors is incorrect. Please see the author list, with the correctly ordered affiliations indicated, and the correctly ordered list of affiliations below.

Mingming Li^2,3^, Xingchang Zhang^1,2^, Qing Zhen^2,3^, Fengpeng Han^1,2^.

1 State Key Laboratory of Soil Erosion and Dryland Farming on the Loess Plateau, Institute of Soil and Water Conservation, Northwest A & F University, Yangling 712100, PR China

2 Institute of Soil and Water Conservation，Chinese Academy of Sciences and Ministry of Water Resources, Yangling 712100, PR China 

3 University of Chinese Academy of Sciences, Beijing 100049, PR China

